# Integrating databases for research on health and performance in small animals and horses in the Nordic countries

**DOI:** 10.1186/1751-0147-53-S1-S4

**Published:** 2011-06-20

**Authors:** Agneta Egenvall, Ane Nødtvedt, Lars Roepstorff, Brenda Bonnett

**Affiliations:** 1 The Department of Clinical Sciences, Faculty of Veterinary Medicine and Animal Science, Swedish University of Agricultural Sciences, SE-750 07 Uppsala, Sweden; 2 Department of Companion Animal Clinical Sciences, Norwegian School of Veterinary Science, Oslo, Norway; 3 The Department of Anatomy, Physiology and Biochemistry, Unit of Equine Studies, Swedish University of Agricultural Sciences, Box 7046, SE-750 07 Uppsala, Sweden

## Abstract

In a world of limited resources, using existing databases in research is a potentially cost-effective way to increase knowledge, given that correct and meaningful results are gained.

Nordic examples of the use of secondary small animal and equine databases include studies based on data from tumour registries, breeding registries, young horse quality contest results, competition data, insurance databases, clinic data, prescription data and hunting ability tests. In spite of this extensive use of secondary databases, integration between databases is less common. The aim of this presentation is to briefly review key papers that exemplify different ways of utilizing data from multiple sources, to highlight the benefits and limitations of the approaches, to discuss key issues/challenges that must be addressed when integrating data and to suggest future directions. Data from pedigree databases have been individually merged with competition data and young horse quality contest data, and true integration has also been done with canine insurance data and with equine clinical data. Data have also been merged on postal code level; i.e. insurance data were merged to a digitized map of Sweden and additional meteorological information added. In addition to all the data quality and validity issues inherent in the use of a single database, additional obstacles arise when combining information from several databases. Loss of individuals due to incorrect or mismatched identifying information can be considerable. If there are any possible biases affecting whether or not individuals can be properly linked, misinformation may result in a further reduction in power. Issues of confidentiality may be more difficult to address across multiple databases. For example, human identity information must be protected, but may be required to ensure valid merging of data. There is a great potential to better address complex issues of health and disease in companion animals and horses by integrating information across existing databases. The challenges outlined in this article should not preclude the ongoing pursuit of this approach.

## Introduction

### Why databases?

In a world of limited resources, using existing databases in research is a potentially cost-effective way to increase knowledge, given that correct and meaningful results are gained. The databases can provide a better understanding of issues at the population level, without costly and time-consuming primary research. Because research based on databases is generally retrospective in nature, it might be reasonable to re-evaluate the results using prospective designs at a later stage. Both primary and secondary databases, i.e. those constructed specifically for a research purpose or not, respectively, might be used depending on the research question [1]. What constitutes a secondary database can sometimes be hard to judge. For example, in this article pedigree databases will be considered secondary, although their primary purposes may be not only to have control over the pedigree of each animal, but also to analyse hereditary progression, both by conventional and research methods. Some of the databases discussed in this article may have been secondary in the aspect that the data were collected for other purposes, but in order to use them for research the data were entered into a computer database.

### Why Nordic countries?

Relative to the population size the Nordic countries are outstanding in the usage of the secondary data in human medical research. Also the opportunity to use secondary databases for research on health and performance in animals in the Nordic countries has proven to be substantial, as judged by the volume of publications in this area. There appears to be a culture and tradition in the Nordic countries for both collecting and sharing data, exemplified by the extensive use of the Agria insurance database (Sweden), compared to the much more limited use of data from other companies in Europe or North America [2]. Other Nordic examples of the use of secondary small animal and equine databases include studies based on data from tumour registries [3-5], breeding registries [6-13], young horse quality contest results [6,7], competition data [7,8], clinic data [14], prescription data [15] and hunting ability tests [16]. In spite of this extensive use of secondary databases, integration between databases is uncommon.

#### Why integration, what is it and what is needed?

A further advancement from using one single database is to integrate information between databases, which facilitates a broader collection of information regarding the study units. Integration can be defined in various ways. The most basic is that information is combined between databases by merging records at the individual animal (or lower, for example limb) level. This will be referred to as *merging at the individual level* in this manuscript. A special case is when records are merged on a higher level, e.g. aggregated at farm or postal code or breed level, which will be referred to as *merging atgroup-level*. A separate concept is when data are not merged, but data from different sources are directly compared in order to yield fuller understanding of a context. This concept will also be discussed, using the term *combining databases*. A pre-requisite for individual (or group-level) integration is that the individual identifier(s) or “key” variables (e.g. breed, gender, date of birth, postal codes) must be correct and can be utilized for linking across databases, which might explain why such studies are still rare.

### Aim

The aim of this presentation is to briefly review some key papers that exemplify different ways of utilizing data from multiple sources, to highlight the benefits and limitations of the approaches, to discuss key issues/challenges that must be addressed in any attempts to integrate data and to suggest future directions.

## Examples from the literature

### Combining databases

Information on reimbursed cases of dystocia and caesarean section in a Swedish animal insurance-database [17] during 1995 to 2002 (n=195,931 insured bitches) were combined with information from the Swedish Kennel Club [18] registrations on litters and puppies (n=81,306 litters) born during the same time. Incidence of dystocia among insured Swedish bitches was calculated using the insured population. Furthermore, the proportion of all whelpings that yielded a claim for dystocia was estimated by combining information from the two databases. No formal merging of the databases was performed, and the combined results should be interpreted with caution. However, the two populations are both considered representative of the Swedish dog population where ~30% of dogs are covered by an insurance plan with Agria and >90% of pure-bred dogs are registered with the SKC [19].

In a Norwegian publication, information from a population-based tumour registry, the Norwegian Canine Cancer Register, was combined with a census among all owners of dogs from the three breeds boxer, bichon frisé and Bernese mountain dog (n=5992 females at risk) [20]. Several methods were used to find all tumours, e.g. skin lumps, removed from the dogs during this period. For example each clinic in four counties signed a written agreement to submit all these to this specific registry and during this time all pathology examinations were free of charge.

Based on these two databases both crude and age-specific incidence rates of mammary tumours for the three breeds were calculated. The return-rate for the census which gave rise to the age-distribution among the bitches was 70%, which must be considered good. Potential problems or bias related to the merging or to the assumptions that were made about rate of death in the population were not discussed in the paper [20].

### Integrating databases

#### Merging at group-level

Breed-level merging has been performed when estimating the relative risk of selected canine cancers [3]. Histologically verified canine tumours : (n=14,401 tumours) from the Norwegian Canine Cancer Register between 1990 and 1998 were used as numerator, and merged by breed to the number of dogs registered with the Norwegian Kennel Club [21]between 1982 and 1997, which was the denominator. The resulting relative risk was presented for five specific tumours among breeds with more than 50 registered tumours of all kinds during the study period [3]. It is noted that the method is uncertain because the knowledge about the population size is approximate only.

The geographical variation in the incidence rate of canine atopic dermatitis (CAD) in Sweden has been investigated and the association between such variation and selected environmental risk factors evaluated [21]. The unit of analysis was the postal code area (PCA), and information on the incidence rate of CAD from the Agria insurance company was aggregated to this level based on the postal code of the owner's address. The database (n=220, 835 insured individuals with 1,245 with at least one claim for CAD, out of which 1,235 were matched to one of the 559 postal code areas) was merged to a digitized map of Sweden which contained the spatial location of the PCAs as well as the human population density per PCA. Additional meteorological information (average temperature, rainfall and more) was added from the Swedish National Atlas [22]. Information from the three databases was combined and used to visualize and explore spatial relationships in the incidence of CAD, as well as to generate a “risk map” based on the predictions from a generalized linear mixed Poisson-regression model with PCA as random variable [23].

#### Merging at the individual level

Data from three sources; pedigree data [24], young horse quality contest data and competition results [25] were integrated [7]. There were 3,708 Warmblood horses (born between 1968 and 1982) that had participated in the young horse quality contests as 4-year-olds and 25,605 horses (born between 1953 and 1995) with competition records, all of which had an identified pedigree. For dressage and show-jumping 1,206 and 1,879 horses were available for analysis. Any problems found during merging were not mentioned, likely because both the competition database and the RHQT rely heavily on correct pedigree recording, making merging straight forward. In addition, the young horse quality contests were entered manually from written protocols. One problem/benefit discussed regarding the ability to extrapolate the results was that selection bias was considered small because 35% of 4-yr old horses participated in the young horse quality contest [7]. From an epidemiological point of view this proportion can both be considered large or small; large as to actually have attracted many horse owners but too small to have convincingly covered a majority of the population.

Information on five dog breeds from insurance and kennel club data (n>28,000 dogs) was integrated to study hip dysplasia (HD) [12]. The details of the integration was as follows: by breed “between 61% (German Shepherds) and 77% (Rottweilers) of all dogs registered by the Swedish Kennel club with a Swedish registration number born during 1994–2003 had an official screening result for HD”. Among the breeds analysed, 58% of the dogs from the insurance database were included. This can be considered acceptable because not all dogs are screened for HD. Also, “the proportion of dogs with a hip screening result in the SKC data that also had a life and/or veterinary insurance in the edited data from Agria ranged from 36% to 51%”. It was argued that it should be possible to generalise the results to a larger dog population, at least for these breeds. Data identifiers could have been better than they were in the insurance data but checking of the data showed correct merging (results not presented).

Data from one large regional animal hospital and pedigree data were studied relative to osteochondrosis lesions [26]. The data used included information on horses screened (prior to breaking, sale or breeding evaluation; n= 879) for osteochondrosis and other osseus fragments as well as horses examined due to clinical symptoms from the locomotor apparatus (n=3,639). The estimated heritabilities were within the range of results published previously. One reason this study was thought to have good results was that the same radiologist examined all the radiographs, which may of course also lead to bias if this radiologist differed in his/her evaluations from the general consensus. However, 26% of the horses were lost because of lack of valid animal identity- which can be considered a substantial fraction. It was argued that providing routine clinical data for research could be valuable. However, the animals analysed must be an unbiased sample of the population from the analytical aspect. This is most likely to happen when one clinic will be the sole provider of a specific service or if data from several clinics can be combined. This may be a situation more often found in the Nordic countries because of the relatively long distances between clinics. We advise that clinical data not emanating from screening programs should be used even more cautiously.

## Discussion

### What is needed for individual or group-level merging?

A pre-requisite for integration is that the individual identifier(s) must be correct and linkable across databases, which might explain why such studies are still rare. Ideally, merging should be done on a unique indicator for each animal, e.g. registration number or chip number. “Second best” is if, using predefined categories (e.g. breed codes), the merge may be performed using several identifiers. For example, if merging two databases with pedigrees where imperfect identity is found in one of them, the merging by name, breed, year of birth and grandsire is more accurate than merging only on name, although more records may be lost. It may be possible to merge in steps, starting with the merge with most identifiers and successively determining number of individuals merged in each step. Other issues arise when the merge relates to dates as well, i.e. how close events need to be in time to be considered a true match. This may depend on the research question.

Some databases contain unique individual identifiers dating back to the basic construction of the databases, e.g. the genetic databases. The level of detail and quality of this information is closely linked to the original purpose of the database. For example, at breeding evaluation, a unique and exact identity is paramount. Comparing to research on humans in the Nordic countries, where epidemiologic studies have been facilitated by the omnipresent personal identity number, research in companion animals and horses is, in general, hampered by less accurate identification. The possible exception being genetic studies in meticulously controlled breeds or populations. Identities are likely most accurate (and correctly transcribed in all aspects) when recorded electronically and verified each time an individual is encountered or when the very identity is absolutely central to the context. To ensure absolute correct identification of the individual a subcutaneously injected microchip carrying a unique, electronic identification-number is ideal because it actually stays with the animal. However, it seems no system is perfect as, even with the Swedish personal identity numbers there are problems, e.g. relative to immigrants [27]. It should also be borne in mind that outside the Nordic countries personal-identity information is generally less available and its use more sensitive. A recent example of challenges encountered when merging human databases from the US has been provided [28].

### Group-level merging and combining databases

The criteria for merging at group-level are less strict regarding individual identification. Therefore, integration at this level is easier to perform and probably more widely applied. However, loss of detail regarding the study units is inherent in the methodology and the research questions asked will not be the same as for individual level merges. If the groups are crude there may be a risk of ecological fallacy, which means that while data are analysed at group level, the researcher or readers may make inferences to a lower level, e.g. individuals, which may or may not be true. Depending on the topic, the strength of evidence gained from combining databases might be lower and this needs to be reflected in the presentation and interpretation of results.

### Challenges and possibilities

#### Clinical databases for research

Use of case-based clinical data from veterinary teaching hospitals or private clinics has always been limited by unknown base populations, as well as other issues relative to the client, animal and clinical situation. With the increasing computerization of veterinary clinics, the potential for combining data from multiple sources has increased. However, such analyses should be viewed as multiple-site studies, with all the concerns of individual site studies as well as issues of diagnostic consistency across sites, type and amount of data recorded, as well as representativeness of the cases to the base populations. Unfortunately, the ease of combining the data may overshadow these concerns and standardization and validation may not be done. Further, the mechanism of referral bias will operate to some extent whenever data from primary and secondary referral sites or local, regional and specialty practices are combined. Referral bias was recently demonstrated in data for the veterinary medical database (VMDB) in the US [29]. VMDB actually *combines* clinical data from a number of large North-American referral animal hospitals and has been doing so since the 1960s [30]. The potential impact of between-site differences will depend on the type of research question examined.

#### Confidentiality versus data quality

Privacy of information is both an ethical and a legal issue. However there is often a trade-off between preserving anonymity and achieving data quality simply because mistakes in programming will be much more evident and easy to find when identifiers are still present.

#### Data quality

When integrating data, there is risk of further data loss. A trade-off between fully secured internal validity in few individuals and the possibility to generalize to a larger population may take place, and it is important to achieve a reasonable balance. It is likely that databases may differ in quality, i.e. validity and exactness of information, but this is not a huge problem given that errors are random. However, when merging databases even more attention must be paid to validity problems. If risks of differential misclassification are identified this should be investigated and taken into account in the analyses. The possibility of introducing bias when combining databases is evident if many individuals are lost and they are not lost by a random process relative to the research objectives. For example it could be possible to limit a study to some breeds or geographic locations where data validity is shown to be good.

### Promoting collaboration

Sometimes the need of the researchers and the database owners may coincide and therefore enhance data quality. Since the year 2002 each registered dog insured at Agria will have its identity verified directly from the Swedish Kennel Club, resulting in year of birth, name and identity number being taken directly from this database, which will enhance merging to a large degree. If the work is done in collaboration with several database owners, the very results presented to these data base owners may lead to increased quality of data over time and increased access to data. The research in itself may therefore be helpful for the database owners. Merging their data with other data can provide insights on a quantitative level not to be found in any other way. Another advantage is that research using secondary databases may bring stakeholders together- enhancing awareness about both possibilities and limitations.

## Conclusion

In addition to all the data quality and validity issues inherent in the use of a single database, additional obstacles arise when combining information from several databases. Loss of individuals due to incorrect or mismatching identifying information can be considerable. If there are any possible biases affecting whether or not individuals can be properly linked, misinformation may result in addition to the reduction in power. Issues of confidentiality may be more difficult to address across multiple databases. For example, human identity information must be protected, but may be required to ensure valid merging of data. There is a great potential to better address complex issues of health and disease in companion animals and horses by integrating information across existing databases. The challenges outlined in this article should not preclude the ongoing pursuit of this approach.

## Competing interests

The authors declare that they have no competing interests.
